# Improving detection performance of hepatocellular carcinoma and interobserver agreement for liver imaging reporting and data system on CT using deep learning reconstruction

**DOI:** 10.1007/s00261-023-03834-z

**Published:** 2023-02-09

**Authors:** Naomasa Okimoto, Koichiro Yasaka, Masafumi Kaiume, Noriko Kanemaru, Yuichi Suzuki, Osamu Abe

**Affiliations:** Department of Radiology, Graduate School of Medicine, The University of Tokyo, 7-3-1 Hongo, Bunkyo-Ku, Tokyo, 113-8655 Japan

**Keywords:** Liver, Hepatocellular carcinoma, Deep learning, Multidetector computed tomography

## Abstract

**Purpose:**

This study aimed to compare the hepatocellular carcinoma (HCC) detection performance, interobserver agreement for Liver Imaging Reporting and Data System (LI-RADS) categories, and image quality between deep learning reconstruction (DLR) and conventional hybrid iterative reconstruction (Hybrid IR) in CT.

**Methods:**

This retrospective study included patients who underwent abdominal dynamic contrast-enhanced CT between October 2021 and March 2022. Arterial, portal, and delayed phase images were reconstructed using DLR and Hybrid IR. Two blinded readers independently read the image sets with detecting HCCs, scoring LI-RADS, and evaluating image quality.

**Results:**

A total of 26 patients with HCC (mean age, 73 years ± 12.3) and 23 patients without HCC (mean age, 66 years ± 14.7) were included. The figures of merit (FOM) for the jackknife alternative free-response receiver operating characteristic analysis in detecting HCC averaged for the readers were 0.925 (reader 1, 0.937; reader 2, 0.913) in DLR and 0.878 (reader 1, 0.904; reader 2, 0.851) in Hybrid IR, and the FOM in DLR were significantly higher than that in Hybrid IR (*p* = 0.038). The interobserver agreement (Cohen’s weighted kappa statistics) for LI-RADS categories was moderate for DLR (0.595; 95% CI, 0.585–0.605) and significantly superior to Hybrid IR (0.568; 95% CI, 0.553–0.582). According to both readers, DLR was significantly superior to Hybrid IR in terms of image quality (*p* ≤ 0.021).

**Conclusion:**

DLR improved HCC detection, interobserver agreement for LI-RADS categories, and image quality in evaluations of HCC compared to Hybrid IR in abdominal dynamic contrast-enhanced CT.

**Graphical Abstract:**

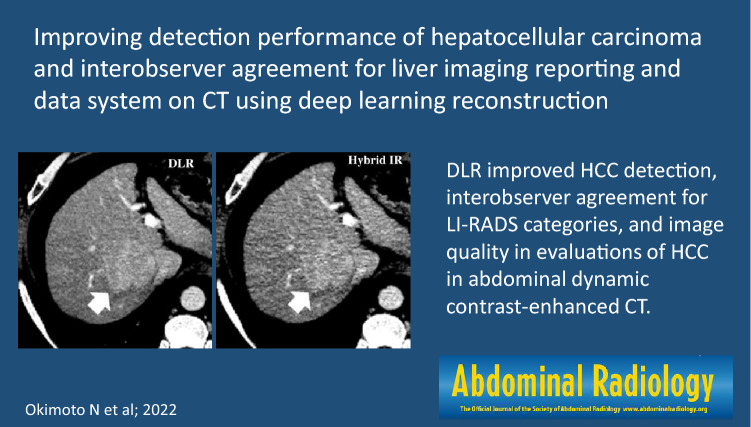

## Introduction

Hepatocellular carcinoma (HCC) is one of the most common cancers and causes cancer-related deaths worldwide [1]. There are multiple treatment options for HCC: surgery, radiofrequency ablation, transplantation, transcatheter arterial chemoembolization, and systemic treatment [2]. In considering the treatment strategy, the size and number of HCCs play an important role. Thus, the accurate diagnosis of HCCs is crucial to determine the appropriate treatment options. Moreover, unlike other tumors, most HCCs can be diagnosed radiographically and treated without invasive biopsy or surgery [3]. The American Association for the Study of Liver Diseases recommends CT or MRI to diagnose HCC [4]. CT is more easily accessible and easier to perform than MRI. Therefore, CT is widely used in daily clinical practice. However, the diagnostic performance of MRI is known to be higher than that of CT [5].

The Liver Imaging Reporting and Data System (LI-RADS) first version was released in 2011 by the American College of Radiology to standardize radiology reporting of HCC in high-risk patients in terms of screening, surveillance, diagnosis, and treatment response assessment, and LI-RADS version 2018 is the latest version [6]. Though one of the key motivations of LI-RADS was to reduce variability in the interpretation of imaging findings [7], a recent study shows that LI-RADS version 2018 had relatively lower inter-reader agreement than previous versions of LI-RADS [8].

The applications of deep learning have been gaining wide attention in the field of radiology [9]. Deep learning has strong presence in not only lesion detection [10, 11] but also differential diagnosis [12] and disease staging [13]. Recent studies have shown that deep learning can also be used for image processing [14]. Deep learning reconstruction (DLR) is one of such algorithms. DLR reduces image noise and improves image quality compared to conventional hybrid iterative reconstruction (Hybrid IR) [15, 16]. Since liver lesion detection on CT is known to be inversely correlated with image noise [17], DLR would have the potential to improve the diagnostic performance of CT in detecting HCC. Inter-reader agreement of LI-RADS may also be improved with the use of DLR. However, while there exist several studies regarding image quality of DLR [15, 16], there are no studies on HCC detection or LI-RADS agreement compared to Hybrid IR.

This study aimed to compare HCC detection, interobserver agreement for LI-RADS categories, and image quality between DLR and Hybrid IR.

## Methods

This retrospective study was approved by our Institutional Review Board, and the requirement for obtaining written informed consent was waived.

### Patients

**Fig. 1:**
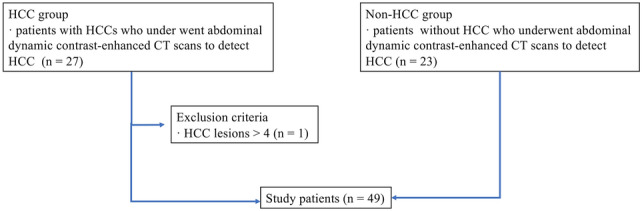
Flowchart of patient inclusion process and image analysis. *HCC* hepatocellular carcinoma

We searched the picture archiving and communication system for all consecutive patients who underwent CT scan for the evaluation of HCC. Figure 1 summarizes the patient inclusion process.

**Fig. 2:**
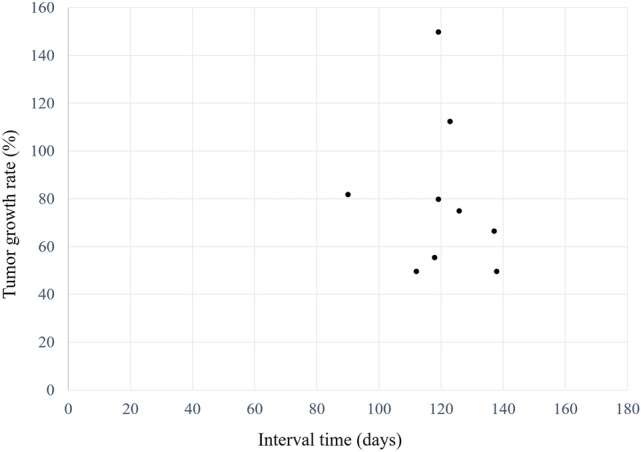
A size change for HCC which showed ≥ 50% size increase in ≤ 6 months. The figure shows tumor growth rate and interval time

For the HCC group, patients who underwent abdominal dynamic contrast-enhanced CT between October 2021 and March 2022 in which one or more HCCs were identified were included in the study. Patients with four or more HCCs were excluded; according to the guideline [18], those patients will have a potential to be treated with systemic treatment and identifying all lesions have little clinical benefit for its burden on radiologists. A total of 26 patients and 42 HCCs were identified. There were also 5 hemangiomas and 2 focal nodular hyperplasias. However, since the main purpose of this study was to evaluate the detection performance of HCCs, these lesions were not evaluated in the following analyses. Two radiologists (A and B with imaging experience of 5 and 12 years, respectively) established the standard for the diagnosis of HCC with reference to the following modalities: histopathological report (8 lesions), CT image findings with chronological change in size (≥ 50% size increase in ≤ 6 months) (9 lesions) (Fig. 2), combinations of CT and MRI image findings (13 lesions), and CT image findings (12 lesions). All lesions were treated with surgery (7 lesions), liver transplantation (1 lesion), or radiofrequency ablation (34 lesions).

The inclusion criterion for the non-HCC group was the absence of HCC on abdominal dynamic contrast-enhanced CT in February and March 2022 (the study period was different between the HCC group and the non-HCC group in order to balance the number of patients for these two groups). The absence of HCC was confirmed based on the following modalities: histopathologically 1 patient who underwent liver transplantation, no chronological change with examinations including MRI over 4 months (3 patients), no chronological change with CT examinations over 4 months (18 patients), and image findings at a single CT examination (1 patient). In consequence, 23 patients met the criterion.

A total of 49 patients (26 patients in the HCC group and 23 patients in the non-HCC group) were included in the final analyses (qualitative image analyses part 1 and 2, and quantitative image analyses, as described later). Table 1 shows age, sex, body mass index, hepatitis B viral status, hepatitis C viral status, the presence of histopathologically proven cirrhosis, and CT dose index volume in the HCC and non-HCC groups.

**Table 1:** Demographic and Clinical Characteristics in the HCC and Non-HCC Groups

	HCC group (*n* = 26)	Non-HCC group (*n* = 23)	*P* value
Age (years: mean ± SD)	73 ± 12.3	66 ± 14.7	0.104^a^
Sex (male, female)	19, 7	11, 12	0.086^b^
Body mass index (kg/m2: mean ± SD)	24.6 ± 4.1	23.3 ± 3.4	0.685^a^
Hepatitis B virus (positive, negative)	5, 21	4, 19	1.000^b^
Hepatitis C virus (positive, negative)	10, 16	5, 18	0.233^b^
Presence of histopathologically proven cirrhosis (positive, negative, no pathological data)	8, 7, 11	7, 4, 12	0.707^b^
CT dose index volume (mGy: mean ± SD)	10.7 ± 3.3	10.2 ± 2.9	0.383^a^

*HCC * hepatocellular carcinoma

^a^Mann–Whitney *U* test

^b^Fisher’s exact test

### CT imaging

All patients underwent CT with a multi-detector row CT (Aquilion ONE; Canon Medical Systems, Otawara, Japan). CT scanning parameters were as follows: tube voltage, 120 kVp; tube current, automatic tube current modulation with SD set at 13.0 Hounsfield units; helical pitch, 0.8125:1; and gantry rotation time, 0.5 s. The concentration and volume of the contrast material were determined based on the body weight: 300 mgI/mL and body weight × 2 mL, respectively, for those weighing < 50 kg; 350 mgI/mL and 100 mL, respectively, for those weighing between 50 and 60 kg; and 370 mgI/mL and 100 mL, respectively, for those weighing > 60 kg. Contrast material was injected via the peripheral vein within 30 s. The arterial, portal, and delayed phase images were scanned with the following delays: arterial phase, using a bolus tracking system (threshold attenuation of 200 Hounsfield units in the descending aorta at the level of the diaphragm; portal phase, 40 s after arterial phase; and delayed phase, 180 s after the beginning of contrast agent injection). From the source data, images were reconstructed with the following algorithms: DLR (AiCE body sharp standard, Canon Medical Systems) and Hybrid IR (AIDR 3D enhanced standard with kernel of FC03, Canon Medical Systems). The following image reconstruction parameters were the same across all the image sets: field of view, 35–40 cm (adjusted to body size), and slice thickness/interval, 3/3 mm.

CT images were anonymized and exported from the picture archiving and communication system in Digital Imaging and Communications in Medicine format.

### Qualitative image analyses

In qualitative image analyses, two other radiologists (readers 1 and 2, with 4 and 7 years of imaging experience, respectively, and reader 2 specialized in abdominal radiology) were involved. Qualitative image analyses comprised two parts: HCC detection test and LI-RADS scoring (part 1) and image quality evaluation (part 2). In both parts, single image set, consisting of arterial, portal and delayed phase, was evaluated at a time. The two readers evaluated the images using Image J (https://imagej.nih.gov/ij/).

#### HCC detection test and LI-RADS scoring (part 1)

In this part, the two readers independently identified HCCs by scoring diagnostic confidence (5, definitely present; 4, probably present; 3, possibly present but uncertain; 2, probably not present; 1, definitely not present) and LI-RADS categories (LR 1 to 5) based on version 2018 [6] and recording the location (CT slice number and liver segment). The two readers were also asked to measure the size of the HCC. LI-RADS was categorized with the evaluations of major features consisting of non-rim arterial phase hyper-enhancement (APHE), nonperipheral washout appearance, enhancing capsule appearance, and size of HCC. However, because previous CT images were not necessarily available in all the patients, the threshold growth was not considered in this study. Ancillary features and tiebreaking rules were applied to upgrade or downgrade category [6], and the final LI-RADS category was used in the analyses.

To avoid overestimating the detection performance in DLR, the two readers were asked to evaluate the DLR image sets in session 1 followed by Hybrid IR image sets in session 2 with 2 weeks wash-out period between the two sessions. The single session was performed within a single day. They were blinded to the image reconstruction algorithm. Furthermore, they were not informed of the purpose of the study. The order of the image sets within the DLR and the Hybrid IR was randomized by the radiologist A before the readers’ evaluation. The time required to evaluate one image set was also measured.

After completion of the two sessions, the readers were asked to score LI-RADS categories for the missed HCCs (the diagnostic confidence was not scored in this process).

#### Image quality (part 2)

After part 1, the two readers independently evaluated the image sets, in terms of the following:

- Depiction of major features of HCC on LI-RADS (APHE, nonperipheral washout, and enhancing capsule) (5, clear depiction; 4, clearer than standard; 3, standard; 2, blurred than standard; and 1, very blurred).
- Subjective image noise for the arterial, portal, and delayed phases separately on a 5-point scale (5, almost no noise; 4, less than standard noise; 3, standard noise; 2, more than standard noise; and 1, severe noise).
- Image quality on a 5-point scale (5, excellent; 4, better than standard; 3, standard; 2, worse than standard; 1, poor).

In this part, all image sets (including both the DLR and Hybrid IR) were randomized by radiologist A before the evaluation by the two readers. The two readers evaluated images of one reconstruction algorithm at a time (i.e., not in a side-by-side way). The two readers were also blinded to the image reconstruction algorithm.

### Quantitative image analyses

The radiologist A placed regions of interest with the size of approximately 100 mm^2^ on the abdominal aorta at the level of celiac artery origin. The SD of the CT attenuation, which is an indicator of image noise, was recorded in the arterial, portal, and delayed phases. These evaluations were also performed with Image J (https://imagej.nih.gov/ij/).

### Statistical analysis

Statistical analyses were performed with EZR version 4.0.0 (https://www.jichi.ac.jp/saitama-sct/SaitamaHP.files/statmed.html) [19], which is a graphical user interface of R version 4.2.0 (https://www.r-project.org/) (R Foundation for Statistical Computing, Vienna, Austria).

Fisher’s exact test and the Mann–Whitney *U* test were used to compare the demographic and clinical characteristics in the HCC and non-HCC groups.

The results for continuous variables and ordinal scales were compared between DLR and Hybrid IR with the paired t test and Wilcoxon signed-rank test, respectively, except for the comparison for the depiction of enhancing capsule, for which the Mann–Whitney *U* test was performed. To assess the diagnostic performance in detecting HCCs with the diagnostic confidence score, jackknife alternative free-response receiver operating characteristic analysis was performed with R package of “RJafroc,” and the figures of merit (FOM), which corresponds to the area under the curve in the conventional receiver operating characteristic analysis, were calculated. In calculating the sensitivity for the detection test, diagnostic confidence scores of 3 or more indicated positivity for the presence of lesions. The sensitivities were compared between DLR and Hybrid IR with McNemar’s test. For these comparisons, a *P* value < 0.05 considered statistically significant.

For the LI-RADS categories, interobserver agreement between the two readers was evaluated with the Cohen’s weighted kappa analysis (quadratic weight was used). Based on the Cohen’s article [20], 95% CI for the kappa value was also calculated. Kappa values of 0.00–0.20, 0.21–0.40, 0.41–0.60, 0.61–0.80, and 0.81–1.00 indicated poor, fair, moderate, good, and excellent agreement, respectively.

## Results

### Qualitative image analyses

#### HCC detection test and LI-RADS scoring (part 1)

**Fig. 3:**
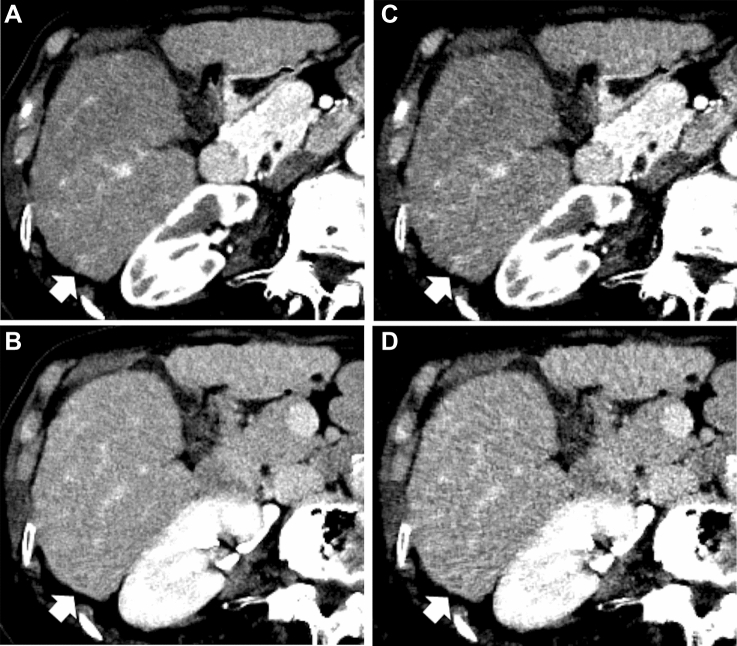
82-year-old woman with hepatocellular carcinoma (HCC) (arrows). **A**–**D** Axial dynamic contrast‐enhanced CT images reconstructed with deep learning reconstruction (DLR) (**A**, **B**) and hybrid iterative reconstruction (Hybrid IR) (**C**, **D**) in the arterial phase (**A**, **C**) and delayed phases (**B**, **D**). Reader 1 identified HCC with DLR (**A**, **B**) but missed it with Hybrid IR (**C**, **D**). Reader 2 missed the HCC in both the DLR and Hybrid IR. The depictions of arterial phase hyper-enhancement, nonperipheral washout, enhancing capsule, image noise in the arterial and delayed phases, and overall image quality were 4/2/2/4/4/3 with DLR and 2/2/-(enhancing capsule was not identified)/2/2/3 with Hybrid IR by reader 1 and 3/3/3/4/4/4 with DLR and 3/3/3/3/3/3 with Hybrid IR by reader 2, respectively

**Fig. 4:**
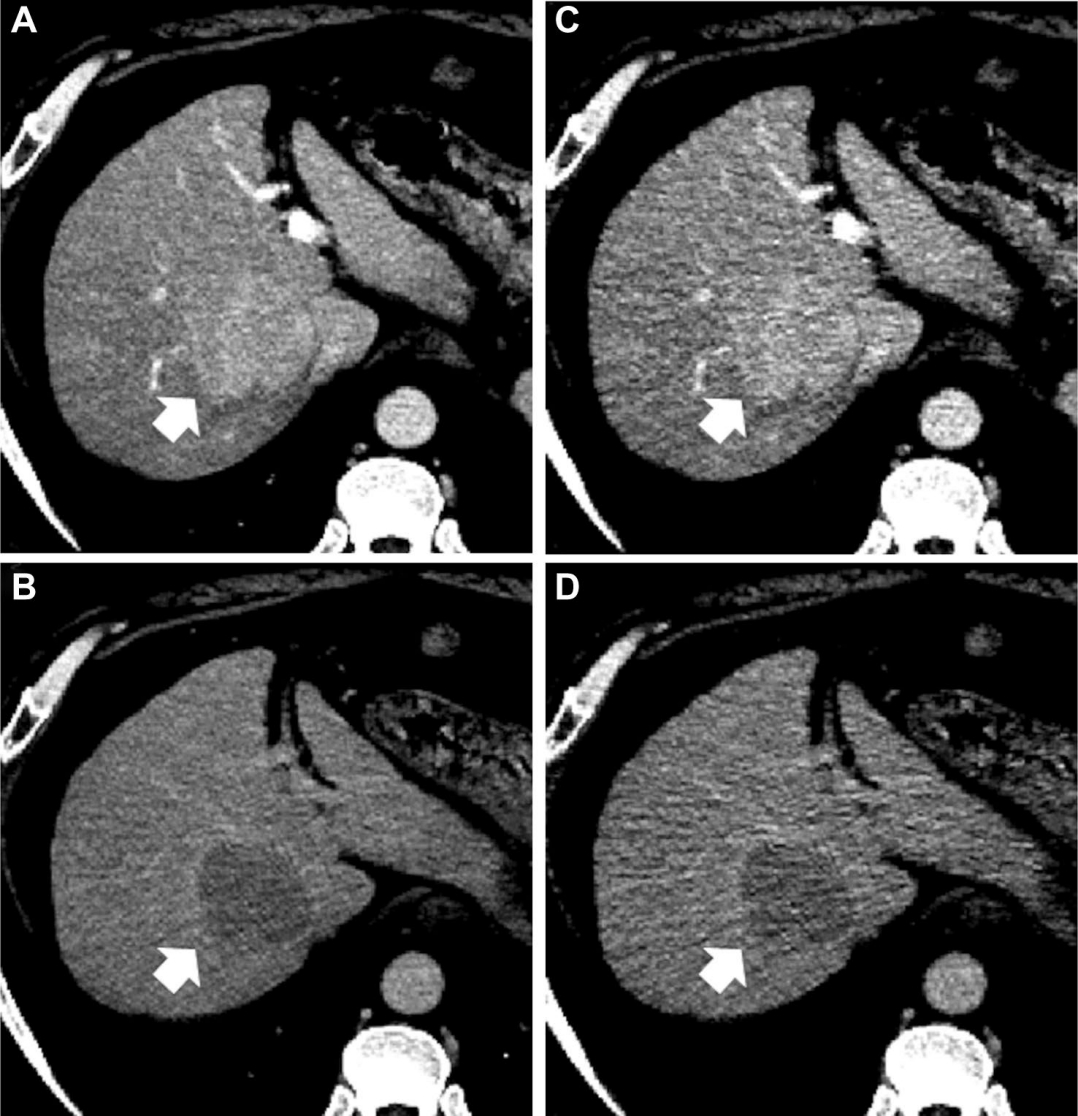
57-year-old man with hepatocellular carcinoma (HCC) (arrows). **A**–**D** Axial dynamic contrast‐enhanced CT images reconstructed with deep learning reconstruction (DLR) (**A**, **B**) and hybrid iterative reconstruction (Hybrid IR) (**C**, **D**), in the arterial phase (**A**, **C**) and delayed phase (**B**, **D**). Readers 1 and 2 identified the HCC with both DLR (**A**, **B**) and Hybrid IR (**C**, **D**). The depictions of arterial phase hypermage quality were rated as 4/4/4/4/4/4 with DLR and 2/2/2/2/2/2 with Hybrid IR by reader 1 and 3/3/3/3/3/3 with DLR and 3/3/3/3/3/3 with Hybrid IR by reader 2, respectively

The detailed results of the HCC detection test and interobserver agreement analyses for LI-RADS scores are shown in Table 2. Representative CT images are shown in Figs. 3 and 4.

**Table 2:** Results for qualitative image analyses: HCC detection test and interobserver agreement for LI-RADS categories

	DLR	Hybrid IR	*P* value
HCC detection			
Figure of merit (based on reader’s diagnostic confidence)^a^			
Reader 1	0.937	0.904	
Reader 2	0.913	0.851	
Mean (95% CI)	0.925 (0.882–0.968)	0.878 (0.813–0.942)	0.038
Interobserver agreement for LI-RADS^b^			
Kappa (95% CI)	0.595 (0.585–0.605)	0.568 (0.553–0.582)	
Size (mm) (mean ± SD)^c^			
Reader 1	17.4 ± 13.8	17.8 ± 13.9	0.649
Reader 2	18.7 ± 13.7	18.1 ± 13.8	0.211
Reading time per image set (s) (mean ± SD)^c^			
Reader 1	148 ± 53.4	135 ± 86.2	0.268
Reader 2	148 ± 95.5	165 ± 117	0.422

*DLR* deep learning reconstruction, *Hybrid IR* hybrid iterative reconstruction

^a^Jackknife alternative free-response receiver operating characteristic analysis

^b^Cohen’s weighted kappa statistics

^c^Paired *t* test

**Fig. 5:**
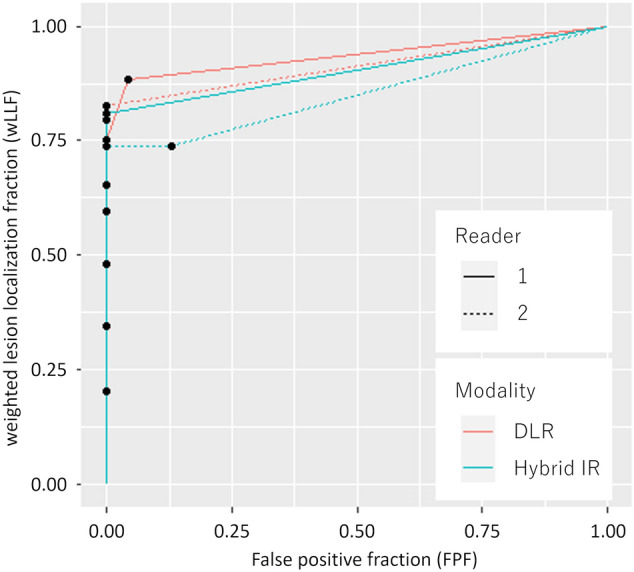
Free-response receiver operating characteristic curve for the detection of hepatocellular carcinoma. Figures of merit were 0.937 and 0.913 for readers 1 and 2, respectively, in DLR and 0.904 and 0.851 for readers 1 and 2, respectively, in Hybrid IR. *DLR* deep learning reconstruction, *Hybrid IR* hybrid iterative reconstruction

The FOM, the diagnostic performance in detecting HCCs, averaged for the readers were 0.925 (reader 1, 0.937; reader 2, 0.913) in DLR and 0.878 (reader 1, 0.904; reader 2, 0.851) in Hybrid IR (Fig. 5). DLR performed significantly better than Hybrid IR for the detection of HCCs (*p* = 0.038). The sensitivities in detecting HCCs were 83% and 79% for readers 1 and 2, respectively, for DLR (Table 3). Though there was no statistically significance, these values tended to be superior to those for Hybrid IR (76% and 69% for readers 1 and 2, respectively). The sensitivities in detecting small HCC were 60–67% and 47–60% in DLR and Hybrid IR, respectively, for those with < 10 mm, and 80–87% and 73–80% in DLR and Hybrid IR, respectively, for those with 10–20 mm. The numbers of false positive findings were 5 and 0 for readers 1 and 2, respectively, for DLR and 5 and 6 for readers 1 and 2, respectively, for Hybrid IR. All detected false positive findings corresponded to the arterioportal shunt, because of no washout in delayed phase and no significant changes on previous and/or follow-up imaging studies.

**Table 3:** Sensitivity (%) in Detecting HCCs

	Reader 1			Reader 2		
	DLR	Hybrid IR	Comparison (*P* values)	DLR	Hybrid IR	Comparison (*P* values)
< 10 mm	67 (10/15)	60 (9/15)	1.000	60 (9/15)	47 (7/15)	1.000
10–20 mm	87 (13/15)	80 (12/15)	1.000	80 (12/15)	73(11/15)	1.000
≥ 20 mm	100 (12/12)	92 (11/12)	N/A	100 (12/12)	92 (11/12)	N/A
All	83 (35/42)	76 (32/42)	0.683	79 (33/42)	69 (29/42)	0.683

Comparisons were performed using McNemar’s test

*DLR* deep learning reconstruction, *Hybrid IR* hybrid iterative, *N/A* not applicable

The results of interobserver agreement for LI-RADS categories are summarized in Table 2. The interobserver agreement was moderate for DLR (0.595; 95% CI, 0.585–0.605) and also moderate for Hybrid IR (0.568; 95% CI, 0.553–0.582). Because there was no overlap between the 95% CIs, the interobserver agreement for DLR was considered to be significantly superior to that for Hybrid IR.

**Fig. 6:**
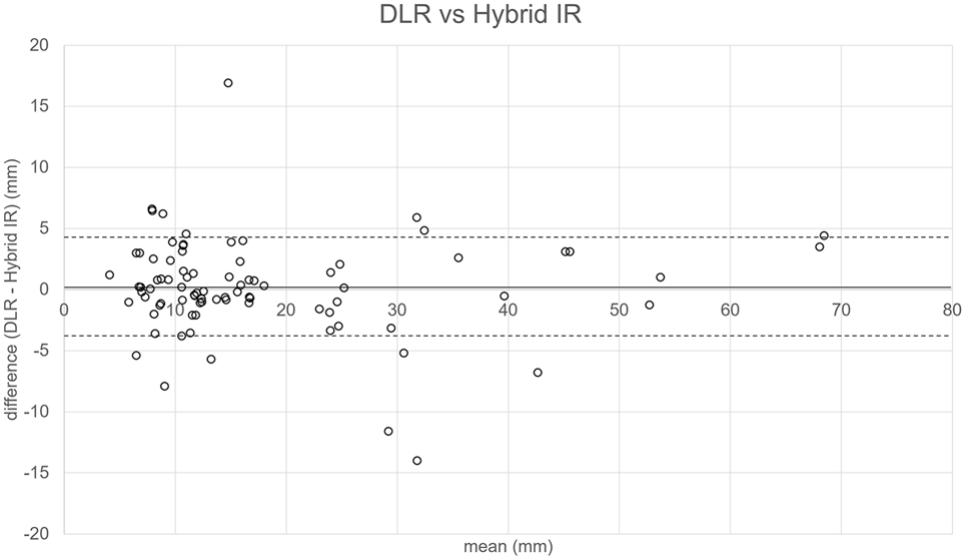
The Bland–Altman plot for the size of hepatocellular carcinoma measured by the two readers. The solid and dotted lines denote the bias (0.19 mm) and lower/upper limit of agreement (− 3.78/ 4.17 mm), respectively. *DLR* deep learning reconstruction, *Hybrid IR* hybrid iterative reconstruction

The Bland–Altman plot for the size of the HCCs (pooled for the readers 1 and 2) is shown in Fig. 6. The bias (with 95% CI) and limit of agreement (with lower limit–upper limit) for the difference of the two readers’ measurements were 0.19 (− 0.66–1.04) mm and 3.97 (− 3.78–4.17) mm, respectively.

#### Image quality (part 2)

The detailed results of the qualitative image analyses are shown in Table 4. Both readers agreed that DLR was significantly superior to Hybrid IR in terms of depiction of each major feature of HCC (*p* ≤ 0.021), noise in each phase (*p* < 0.001), and overall image quality (*p* < 0.001).

**Table 4:** Results for qualitative image analyses: image quality

	Reader 1			Reader 2		
	DLR	Hybrid IR	Comparison (*P* values)	DLR	Hybrid IR	Comparison (*P* values)
Depiction of major features of HCC (score 5/4/3/2/1)						
Enhancement	13/11/2/0/0	0/0/21/5/0	< 0.001^a^	1/17/8/0/0	0/3/18/5/0	< 0.001^a^
Washout	13/8/2/3/0	0/0/16/10/0	< 0.001^a^	0/1/14/11/0	0/10/13/2/1	0.002^a^
Capsule	7/5/0/3/0	0/0/8/5/0	0.002^b^	0/2/5/0/0	0/0/4/4/0	0.021^b^
Image noise (score 5/4/3/2/1)						
Arterial phase	26/21/2/0/0	0/0/41/8/0	< 0.001^a^	5/38/6/0/0	0/2/38/9/0	< 0.001^a^
Portal phase	26/21/2/0/0	0/0/41/8/0	< 0.001^a^	11/31/6/1/0	0/1/40/8/0	< 0.001^a^
Delayed phase	26/21/2/0/0	0/0/41/8/0	< 0.001^a^	4/39/6/0/0	0/0/41/8/0	< 0.001^a^
Overall image quality (score 5/4/3/2/1)						
	25/21/3/0/0	0/0/41/7/1	< 0.001^a^	4/39/6/0/0	0/0/39/10/0	< 0.001^a^

Numbers of patients for each score are shown

*HCC* hepatocellular carcinoma, *Hybrid IR* hybrid iterative reconstruction, *DLR* deep learning reconstruction

Comparisons were performed using the ^a^Wilcoxon signed-rank test and ^b^Mann–Whitney *U* test

### Quantitative image analyses

The quantitative image noises (i.e., SD of CT attenuations of abdominal aorta) (mean ± SD) were 9.1 ± 2.5 and 11.8 ± 2.7 in the arterial phase, 8.6 ± 0.9 and 11.1 ± 1.8 in the portal phase, and 8.4 ± 1.0 and 10.9 ± 1.7 in the delayed phase for DLR and Hybrid IR, respectively. The quantitative image noise was statistically significantly reduced in DLR than that in Hybrid IR for all phases (*p* < 0.001 for all).

## Discussion

Detection of HCCs is affected by image noise with CT [17], which would have been one of the reasons for inferior detection performance compared to MRI [21]. This study revealed that DLR successfully reduced image noise (*p* < 0.001), which were associated with improved depiction of HCCs compared with Hybrid IR in abdominal dynamic contrast-enhanced CT (*p* ≤ 0.021). Moreover, DLR helped radiologists to detect HCC with a significantly higher performance (FOM of 0.925 for DLR and 0.878 for Hybrid IR, *p* = 0.038) and to score LI-RADS categories with an improved interobserver agreement (0.595 [95% CI, 0.585–0.605] for DLR and 0.568 [95% CI, 0.553–0.582] for Hybrid IR).

Previous studies have reported an improvement in image quality with DLR compared with conventional reconstruction algorithms, such as Hybrid IR [22, 23]. Our results were in line with those reports. Image noise was proven to be reduced in DLR compared to Hybrid IR with both qualitative and quantitative analyses. This would have resulted in significantly clearer conspicuity of HCCs in terms of APHE, nonperipheral washout, and enhancing capsule. To the best of our knowledge, no reports have investigated the conspicuity of HCC on DLR images.

According to a previous study, the diagnostic performance and sensitivity in detecting HCCs with dynamic CT were 0.77 and 58–64%, respectively [21]. In our study, the FOM and sensitivity in Hybrid IR were 0.878 (95% CI, 0.813–0.942) and 69–76%, which were comparable to or slightly higher than those values. The use of DLR improved the diagnostic performance and sensitivity of readers to 0.925 (95% CI, 0.882–0.968) and 79–83%, respectively, exceeding those reported in the previous report. As for the sensitivity in detecting small HCCs, the sensitivities were 47–60% and 73–80% for those with < 10 mm and 10–20 mm, respectively, in Hybrid IR, which were comparable to the sensitivity of 46–56% for HCCs with < 20 mm in the previous report [21]. The sensitivities in detecting small HCCs with DLR (60–67% and 80–87% for those with < 10 mm and 10–20 mm, respectively) also tended to be superior to those in Hybrid IR and the previous study [21]. These findings might result from the improved depiction of major features of HCC in DLR compared to Hybrid IR as evidenced by our study.

There are some HCC treatment options. The tumor stage of HCC, which can be evaluated with CT, plays an important role in determining the treatment strategy. Surgery is selected for patients with early-stage HCC, and the drugs, such as small molecule targeted drugs, are usually used for patients with advanced HCC [2, 24]. DLR may have the potential to improve patient prognosis by providing appropriate treatment strategies to patients and detecting HCC with earlier stages.

LI-RADS describes the standardized imaging features of HCC and criteria for ordinal categories (from LR 1 to 5). LI-RADS was introduced to reduce interobserver variability in interpretation of imaging findings [7, 25]. However, according to a recent study, LI-RADS version 2018 had relatively lower inter-reader agreement (kappa = 0.53) than previous versions of LI-RADS (kappa = 0.69 and 0.79 for LI-RADS version 2014 and 2017, respectively) [8]. The interobserver agreement for LI-RADS version 2018 in Hybrid IR (kappa = 0.568 [95% CI, 0.553–0.582]) in our study was in line with the previous study. Our study also revealed that DLR (kappa = 0.595 [95% CI, 0.585–0.605]) could help improve the interobserver agreement for LI-RADS version 2018.

This study has some limitations. First, the reference standard of HCC diagnosis was not established histopathologically for all patients. In daily clinical practice, HCCs are mainly diagnosed with images. Indications for ablation would slightly different across different institutions, and treatment starts without invasive biopsy or surgery for these patients. Therefore, including patients only with histopathological evaluations would be associated with a selection bias. Although we could not conclude that all hepatic lesions were HCC, it is considered clinically acceptable. Second, the study included a relatively small number of participants (*n* = 49). Nevertheless, DLR was significantly superior to Hybrid IR in detection performance and interobserver agreement for LI-RADS scoring between DLR and Hybrid IR. Further studies including a larger number of patients, especially those with small lesions, would be warranted. Finally, each manufacturer’s DLR has subtle difference in algorithms; thus, the results in this study are not necessarily applicable to DLR of other manufacturers.

In conclusion, DLR improved HCC detection performance, interobserver agreement for LI-RADS categories, and image quality in abdominal dynamic contrast-enhanced CT compared to Hybrid IR.

## Abbreviations

APHE: Arterial phase hyper-enhancement
DLR: Deep learning reconstruction
HCC: Hepatocellular carcinoma
Hybrid IR: Hybrid iterative reconstruction
LI-RADS: Liver Imaging Reporting and Data System
